# Neuronal Antibodies and Associated Syndromes

**DOI:** 10.1155/2019/2135423

**Published:** 2019-07-09

**Authors:** Borros M. Arneth

**Affiliations:** Institute of Laboratory Medicine and Pathobiochemistry, Molecular Diagnostics, University Hospital of the Universities of Giessen and Marburg UKGM, Justus Liebig University Giessen, Giessen, Germany

## Abstract

**Introduction:**

Multiple well-recognized conditions, such as Lambert–Eaton myasthenic syndrome (LEMS) and myasthenia gravis (MG), have been associated with neuronal antibodies.

**Materials and Methods:**

A search was performed using Embase, PubMed, and CINAHL. An initial search of each database was conducted using keywords and terms related to the aim of the current review. Additional articles were obtained by examining the reference lists and citations in the selected records.

**Results:**

The studies identified through the search process used different designs and methods to explore neuronal antibodies and associated syndromes. Previous studies have shown that neurological and psychiatric disorders can be mediated and influenced by various antibodies. The identification of autoantibodies can help with the accurate diagnosis of conditions and commencement of early treatment.

**Discussion:**

A review of selected studies identified in the literature implicated that classic anti-neuronal antibodies, such as anti-Ri and anti-Hu, play a role in the development of neurological diseases. More recent studies have indicated that other novel antibodies act on neuronal cell surface antigens to contribute to the development of neurological disorders.

**Conclusion:**

Existing research provides evidence revealing a spectrum of antibodies linked to the development and progression of neurological diseases. However, further antibody testing and studies should be performed to validate the relationship between conditions and antibodies.

## 1. Introduction

Rigorous experimental and animal studies have shown that conditions, such as autoimmune limbic encephalitis and stiff person syndrome, are mediated and influenced by antibodies [1–3]. In most instances, these antibodies are directed towards ion channels and critical membrane receptors that affect transmission in neuromuscular junctions [4, 5]. These antibodies bind extracellular epitopes and cause neurological dysfunction [6–8], and researchers have reported that different antibodies affect the well-being of patients with neurological syndromes. These antibodies target intracellular proteins rather than pathogens and may lead to disease development [9–11]; in addition, these antibodies may contribute to disease progression by causing synaptic dysfunction [12]. It is widely thought that the cytotoxicity of T-cells accounts for the significant loss of neuronal cells in patients [10, 11]. Additionally, T-cells may facilitate the production of the enzyme glutamic acid decarboxylase (GAD) [13, 14], which can be targeted by autoantibodies in patients with autoimmune diseases [15, 16]. This critical process reportedly contributes to the development of different neurological syndromes. This study aimed to examine how neural antibodies contribute to the development and progression of various clinical conditions.

## 2. Materials and Methods

**Figure 1:**
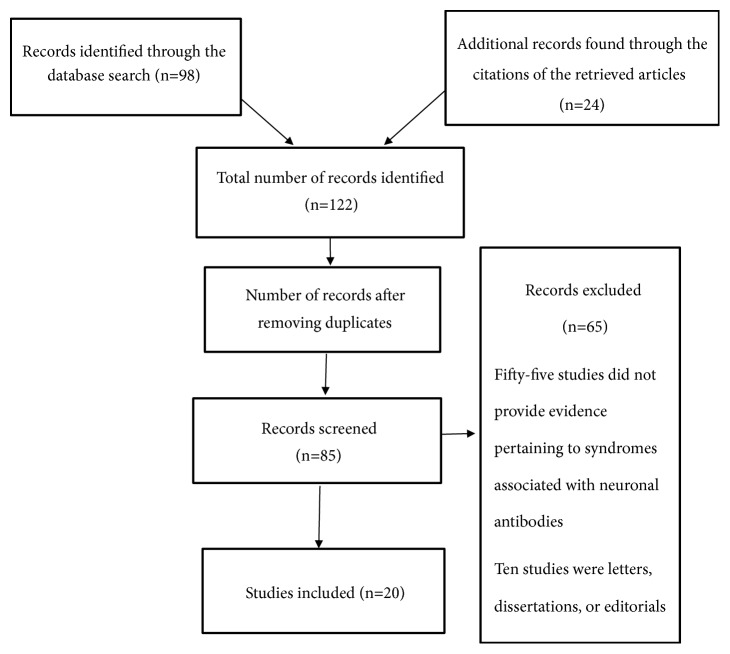
PRISMA flow diagram.

This systematic review intended to examine syndromes linked to neuronal antibodies. This study involved conducting an extensive, systematic search of the literature to locate articles and studies that examined neuronal antibodies and associated syndromes. Additionally, the search focused on identifying studies providing information regarding the mechanism underlying the development of these conditions. The systematic literature search was conducted in 2018, and the primary goal was to identify and analyze peer-reviewed articles related to the study topic. The Embase, PubMed, and CINAHL databases were searched to identify relevant data sources. In each database, the initial search was performed using precise keywords and terms related to the purpose and objectives of the current review, including neuronal antibodies, associated and neurological syndromes, neuroimmunology, diseases, and pathophysiology. In total, 122 studies were identified in the search. After the successful elimination of duplicates from the initial list, 85 records were chosen and subjected to screening to determine their suitability and relevance to the current study. At the end of the screening process, 20 articles met the inclusion criteria. These peer-reviewed studies were used as the basis for the current investigation. For the studies included for investigating autoantibodies associated with neurological disorders see Table 1, for studies investigating antibodies associated with psychiatric disorders see Table 2, for a summary of autoantibodies targets and associated diseases see Table 3 and for the PRISMA flow diagram see Figure 1.

## 3. Results

Neuroimmunology is a relevant and rapidly evolving field. The witnessed changes in this particular area are primarily attributed to the discovery of new syndromes and antibodies. Neurological syndromes are prominent and prevalent in the neuroimmunology literature [15, 17–19]. Furthermore, the significant role of autoantibody-mediated processes in the development of these syndromes has attracted the attention of both researchers and practitioners worldwide [15, 16, 20, 21]. The primary goal of this research was to understand the spectrum of antibodies that contribute to the development of neurological syndromes and develop a phenomenological approach to the categorization, diagnosis, and management of such diseases [10, 17, 20, 22, 23]. Most disorders are categorized as rare conditions, but research evidence shows that they can place a significant burden on individuals and the healthcare sector [18, 24–27]. Antibodies can be detected through the indirect immunofluorescence method [23], which is quite complex as it characterizes autoantibodies not only as negative or positive but also on a scale of positivity, including “half-positive” or “low-positive.” However, there is a consensus among researchers that the early recognition, diagnosis, and management of these disorders are critical for proper recovery [5, 19, 20, 26, 28], protecting patients from adverse effects, and reducing the time of medical treatment. Rapid intervention and therapy are required for the effective management of disorders [16, 29–32].

### 3.1. Neurological Disorders and Autoantibodies

The studies reviewed in this paper examined different neurological syndromes that have been linked to neuronal antibodies. One of the conditions featured in these studies is autoimmune limbic encephalitis. Limbic encephalitis is a condition that encompasses a broad spectrum of complications that usually manifest as epileptic seizures, neuropsychiatric symptoms, and memory deficit [33, 34]. Traditionally, autoimmune limbic encephalitis has been linked to classic paraneoplastic antibodies directed against intracellular neuronal proteins; such antibodies include type 1 antineuronal nuclear antibody (anti-Hu/ANNA-1), ANNA-2, Purkinje cell cytoplasmic antibody type 1 (PCA-1), delta/notch-like epidermal growth factor-related receptor (DNER), amphiphysin, and collapsin response mediator protein 5 (CRMP5) [35–42]. Recent studies have reported a wide range of novel autoantibodies that can contribute to the development of autoimmune limbic encephalitis [43, 44]. These new antibodies differ from the classic antibodies because they are usually directed against antigens at the neuronal cell surface and include antibodies against N-methyl-D-aspartate (NMDA) glutamate receptors, *γ*-aminobutyric acid (GABA) receptors, and voltage-gated potassium channel-associated protein leucine-rich glioma-inactivated 1 (LGI1) [43–48].

Anti-NMDA receptor (NMDAR) encephalitis is another major neurological syndrome linked to neuronal antibodies. NMDAR encephalitis is regarded as an inflammatory encephalopathic autoimmune disorder associated with specific autoantibodies targeting NMDA glutamate receptors [35, 49]. This disease is currently underdiagnosed because of the relatively limited research devoted to this disorder [35, 49]. However, the detection of anti-glutamate receptor (type NMDA) autoantibodies in patients is a primary criterion used in the diagnosis of anti-NMDAR encephalitis [36, 45]. There are several subtypes of this disease with varying symptoms, including seizures, disorientation, memory deficits, and hallucinations [37, 45, 50], that can adversely affect the health and well-being of patients.

Recent research has shown that cerebellar degeneration is a major target of autoimmunity in the central nervous system (CNS) [45] and can have either an insidious or subacute onset. In some instances, cerebellar degeneration is associated with transient neurological symptoms related to spinocerebellar degeneration. The diagnosis of this condition entails conducting tests to identify autoantibodies against targets, such as gliadin, GAD, and TG6 [50]. In addition, cerebellar degeneration has been linked to anti-neural antibodies related to cortical cerebellar atrophy, such as antibodies against metabotropic glutamate receptor 1 (mGluR1). The identification of autoantibodies provides avenues for managing trigger factors, such as gluten and neoplasms, that contribute to cerebellar degeneration.

Neuropathy is a condition characterized by damaged nerves. The signs of neuropathy include numbness and weakness in the hands and feet. Research has revealed serum antibodies against neural antigens in samples obtained from patients with neuropathy [36, 48] of various types, including paraneoplastic neuropathies, monoclonal gammopathy, and inflammatory polyneuropathies. These common autoantibodies, including anti-MAG antibodies, anti-GM1 ganglioside antibodies, and antibodies against neuronal nuclear Hu antigens, have been associated with neuropathies, such as sensory ataxic neuropathy (SAN), acute motor axonal neuropathy (AMAN), and chronic ataxic neuropathy (CANOMAD) [44]. The correlation between neuropathy and the above antibodies suggests a possible avenue for understanding the pathogenesis of each disorder. In addition, these findings have therapeutic implications as these autoantibodies could be targeted to help manage neuropathy [49].

Other researchers have focused on autoantibodies related to the emergence and progression of retinopathy. Retinal degeneration manifests as a sudden or gradual loss of vision and abnormal electroretinography (ERG) potentially caused by the targeting of retinal proteins by autoantibodies [16, 20]. Limited information is available regarding the specificity of the autoantibodies leading to retinal degradation [3]. However, research indicates that in patients with cancer, retinopathy can be associated with the tumor through tumor-induced autoantibodies; for example, such autoantibodies in melanoma lead to melanoma-associated retinopathy (MAR) and, in other forms of cancer, cancer-associated retinopathy (CAR) [51, 52].

Stiff person syndrome is another rare neurological disease investigated in the selected articles. This condition has both nonparaneoplastic and paraneoplastic origins and manifests in patients as severe progressive muscle stiffness in the lower extremities and spine [37]. In the paraneoplastic cases, this condition is linked to antibodies against amphiphysin. In the nonparaneoplastic cases, this illness has been associated with antibodies against GAD, but GAD-associated cases of stiff person syndrome are more common than the paraneoplastic cases [50]. However, notably, anti-GAD antibodies are not regarded as specific and definitive markers of stiff person syndrome because they are present in other neural diseases and complications, such as diabetes mellitus type I [14, 37]. Physicians need to carefully evaluate patients to differentially diagnose paraneoplastic or nonparaneoplastic stiff person syndrome.

Some researchers have reported that autoantibodies may also be involved in the development of dermatomyositis. For instance, anti-Mi-2 antibodies and anti-SRP antibodies have been found in patients recently diagnosed with dermatomyositis [36, 50]. Other autoantibodies associated with this condition include antibodies against nuclear matrix protein 2 (NXP2), histidyl-tRNA synthetase (Jo1), threonyl-tRNA synthetase (PL7), alanyl-tRNA synthetase (PL12), and isoleucyl-tRNA synthetase (OJ) [53–55]. The detection of these autoantibodies in serum samples from patients indicates their possible pathogenic role in the development of dermatomyositis [48, 53–55]. Furthermore, this information can further the development of protocols for the diagnosis and treatment of dermatomyositis [55].

Another group of conditions that has been studied is paraneoplastic neurological disorders (PNDs) [38–40], which affect both the peripheral and central nervous systems and are directly related to tumor development [1–10]. The symptoms of PNDs include brain inflammation, weakness of the hands and feet, progressive numbness, and myoclonus [10]. Research suggests that PNDs are not directly caused by tumor swelling [41, 42]; instead, cancerous cells often express antigens that can induce the formation of specific antibodies associated with paraneoplastic neurological syndrome (PNS). In most cases, the onconeural antibodies found in PNS patients are directed against various neuronal antigens, such as SRY-box 1 (SOX1), Ma2/Ta, PCA-2, CV2, and paraneoplastic antigen MA1 (PNMA1) [41, 42, 46, 56]. The detection of antineuronal antibodies is regarded as sufficient for the diagnosis of PNS [41, 46, 56]. The early detection of these antibodies can help caregivers recognize PNS during the early stages and develop plans that could help manage its impact on patient health and well-being [11–14].

Celiac disease (CD) is another condition that has been linked to neural antibodies. This autoimmune disorder is often triggered by the ingestion of gluten [36] and can affect a broad range of organs and tissues, including muscles, the nervous system, joints, and the skin. The multisystemic nature of CD has been linked to the diverse locations of associated antigens [2]. The primary autoantigens of CD are tissue transglutaminase type 2 (TG2), TG3, and TG6. In some cases, patients with CD may also suffer from neurological complications, such as dementia, migraines, and multiple sclerosis. Moreover, approximately 8 percent of patients with CD may develop neurological autoantibodies [2, 36]. The data indicate that CD affects fewer patients than other neurological complications, such as autoimmune limbic encephalitis [2, 41].

Opsoclonus-myoclonus, Lambert-Eaton myasthenic syndrome (LEMS), myasthenia gravis (MG), and neuromyotonia (NMT) are also associated with neural antibodies. Opsoclonus-myoclonus occurs due to damage to the cerebellum and is linked to the expression of TG2, TG3, and TG6 [45]. In contrast, LEMS is caused by the disruption of nerve impulses in neuromuscular junctions, while MG is linked to acetylcholine receptor alterations [45]. Finally, NMT occurs when muscle fiber function is compromised due to antibodies related to inflammation. Although rare, these disorders can make it difficult for patients to live a normal life.

### 3.2. Psychiatric Disorders and Autoantibodies

Accumulating research suggests that autoantibodies and receptors found on the surface of neurons can affect the development of psychiatric conditions [46]. In addition, recent studies have suggested that autoantibodies are useful for the recognition of the symptoms of these diseases and suggest new opportunities for the development of treatment strategies [46]. Genetic analysis studies have revealed a wide range of gene variants that affect the risk and progression of psychiatric diseases, such as cognitive and affective dysfunction, Hashimoto's encephalopathy, and schizophrenia [46].

Recent genomic investigations and analyses have suggested that autoantibodies and receptors, such as calcium voltage-gated channel subunit alpha 1C (CACNA1C) and calcium voltage-gated channel auxiliary subunit beta 2 (CACNB2), are among the primary risk factors for psychotic disorders, major depressive disorder (MDD), autism spectrum disorder (ASD), attention-deficit/hyperactivity disorder (ADHD), and obsessive-compulsive disorder (OCD) [57]. In other studies, gene variants in human leukocyte antigen (HLA) locations were shown to increase the risk of autoimmune diseases, such as MDD and ADD. Deficiency in the HLA complement component 4B (C4B) gene has been associated with the risk of dyslexia, ADHD, and ASD [46, 57]. Similar conclusions have been reported for HLA DRB1, which has been linked to the risk of ASD and schizophrenia [58]. These findings suggest that autoimmunity and neuroinflammation play a potential role in the development and progression of different types of psychiatric conditions.

The autoantibodies linked to psychiatric disorders can affect neurodegeneration and neuroinflammation processes [57]; these autoantibodies target proteins, ion channels, and receptors that influence the development of such conditions [58]. In addition, these antibodies eliminate or suppress autoimmune responses linked to the emergence and development of psychiatric disorders.

## 4. Discussion

The current review reveals a spectrum of antibodies linked to the development and progression of neurological diseases [59]. The collective understanding of the association between such conditions and relevant antibodies has expanded in recent years due to advances in testing methods and technology [59, 60]. Studies have shown that classic anti-neuronal antibodies, such as anti-Ri and anti-Hu, play a critical role in the development of neurological diseases and the subsequent symptoms experienced by patients [8, 41, 47]. Recent research has indicated that other novel antibodies act on neuronal cell surface antigens, thereby contributing to the development of neurological diseases [10]. Therefore, scientists contend that the identification of antibodies, such as those against NMDA glutamate receptors, is critical for the diagnosis of neurological diseases [23]. The successful identification of antibodies can assist doctors in diagnosing autoimmune disorders and initiating timely treatment.

Existing research further shows that autoantibody screening has evolved to become a vital tool in the diagnosis and subsequent management of neurological diseases. This method is both fast and reliable and involves the use of indirect immunofluorescence and multiparametric indirect immunofluorescence test (IIFT) systems that entail recombinant cell substrates and mosaics of tissue sections to ensure accurate results [23]. However, in some cases, researchers use immunoblot-based methods with purified antigen panels to confirm the antibody specificity [40]. These results further demonstrate the critical connection between neurological diseases and neural antibodies. Regarding reliability, the lack of clarity in the results obtained from laboratory methods is an issue. For instance, immunofluorescence can yield results on a sliding scale, including positivity with no significant clinical meaning [39]. In such cases, it may be necessary to use different tests to ascertain the presence of particular antibodies.

Various therapeutic approaches have applied knowledge regarding autoantibody-related disorders to improve patient well-being. However, the success of some interventions has been limited due to the complex nature of these diseases. A broad spectrum of therapies focus on the significance of T-cell transmitted autoimmunity when managing deleterious diseases, such as CD. Some drugs used in the management of autoimmune disorders, such as interferon-*β*, were developed on the basis of the understanding of the role of neural antibodies in the emergence and progression of these conditions [32]. Notably, the distribution and location of the autoantigens can affect the success of therapeutic approaches. Recent observations have revealed that autoantibodies targeting surface-level antigens appear to be more susceptible to therapeutic agents than those targeting intracellular antigens [36]. Further investigations are needed to understand how specific agents can be used to improve the well-being of patients suffering from conditions linked to neural antibodies. In addition, practitioners should examine the effect of each strategy implemented on the health of their patients.

## 5. Conclusion

The field of immune-mediated CNS diseases has attracted the attention of researchers in recent years. This particular field is not only exciting but also challenging as it requires intense research investigating these immunotherapy-responsive conditions. This study aimed to examine how neural antibodies contribute to the development and progression of different clinical conditions. This review shows that immunotherapy responses in patients with neurological diseases indicate the involvement of antibodies in the development and progression of these diseases. Knowledge of these processes has been used as the basis for developing interventions and drugs that could lead to optimal health outcomes. Autoantibodies are important and could be of great use in the future. Further antibody testing and studies should be performed to validate the connection between conditions and antibodies and determine how these connections can be used for diagnostic purposes.

## Figures and Tables

**Table 1:** Studies Investigating Autoantibodies Associated with Neurological Disorders.

Citation	Study Purpose	Design	Findings
Lai et al. 2010 [15]	To examine the relationship between LGI1 and limbic encephalitis.	Case series	This study identified potassium channels as critical elements in the association between LGI1 and limbic encephalitis.

Lancaster et al. 2011 [16]	To investigate Caspr2 and the development of encephalitis and NMT.	Systematic review	Caspr2 is a critical autoantigen involved in encephalitis and NMT.

Balint et al. 2015 [17]	To examine the genetic and neurological bases of dystonia syndromes.	Systematic review	Dystonia syndromes occur due to a combination of factors that can compromise neurological system function.

Armangue et al. 2014a [18]	To explore the links among brain autoimmunity, Herpes simplex virus, and encephalitis.	Systematic review	Herpes simplex virus can trigger brain autoimmunity and contribute to the development of encephalitis.

Arino et al. 2014 [19]	To study the effect of cerebellar ataxia and GAD antibodies on the development of neurological disorders.	Systematic review	The study revealed that the immunologic profile of cerebellar ataxia and GAD antibodies contributes to the development of neurological diseases.

Lancaster 2016 [33]	To explore the development, diagnosis, and treatment of autoimmune encephalitis.	Systematic review	Autoimmune encephalitis leads to deficits in cognition and memory. The autoantibody testing showed the involvement of different types of autoimmune responses in the development of this condition.

Berger, Hottenrott, Rauer, Stich 2017 [34]	To investigate the prevalence of onconeural antibodies predicting paraneoplastic etiology.	Retrospective cohort study	All patients were negative for antibodies targeting intracellular onconeural antigens, including PNMA1, PNMA2, Zic4, CRMP5, and SOX1.

Liu et al. 2017 [35]	To explore the clinical course of NMDAR encephalitis.	Systematic review	NMDAR encephalitis is a potentially lethal autoimmune disorder characterized by neurologic and psychiatric symptoms. Anti-NMDAR antibodies play a critical pathogenic role in the development of this condition.

Kim et al. 2014 [36]	To examine pediatric autoimmune encephalitis cases based on anti-neuronal antibody tests.	Randomized controlled trial	In total, 23 cases were included in this study. Eight patients tested positive for the anti-NMDAR antibody, and an additional patient tested positive for the anti-CASPR2 antibody.

Murinson and Guarnaccia 2008 [37]	To examine the distinguishing clinical features of amphiphysin Ab-associated stiff person syndrome.	Longitudinal study	In a sample population of 621 patients, 116 patients had GAD antibodies, while another 11 patients had amphiphysin antibodies.

Panzer and Dalmau [38]	To explore immune-mediated movement disorders with an emphasis on treatment, novel antigens, and clinical–immunological associations.	Systematic review	This study showed that movement disorders are usually immune-mediated. Recognition of clinical–immunological associations in these disorders helps with their diagnosis and successful treatment.

Grant and Graus 2009 [39]	To examine the development, progression, and treatment of paraneoplastic movement disorders.	Systematic review	This study showed that paraneoplastic movement disorders are rare conditions caused by nonmetastatic autoimmune complications and are associated with different serum antibodies, such as those targeting mGluR1, Ta, Tr, PCA-2, ANNA-3, and VGCCA.

Psimaras, Carpentier, and Rossi 2010 [40]	To examine a wide range of paraneoplastic patients and characterize alterations in CSF.	Longitudinal study	The researchers found abnormal CSF in 93 percent of the patients. Additionally, an elevated number of cells were reported in 47 percent of the patients before the third month.

Rakocevic G, Floeter MK 2012 [41]	To examine the clinical spectrum, neurophysiological mechanisms, and treatment options for stiff person syndrome.	Systematic review	This study showed that stiff person syndrome is often idiopathic and related to antibodies against GAD and other proteins that impair GABA synthesis.

Jung, Jeong, Kim, Kim, and Jeon 2014 [42]	To explore cases of stiff person syndrome with favorable outcomes.	Case study	This study reported that stiff person syndrome is a rare disorder often characterized by spasms and fluctuating muscular rigidity. This condition is often associated with antibodies against GAD.

**Table 2:** Studies Investigating Autoantibodies Associated with Psychiatric Disorders.

Citation	Study Purpose	Design	Findings
Jiwon and Levy, 2012 [43]	To review the recent literature related to neuromyelitis optica.	Systematic review	This study showed that neuromyelitis optica is a recurrent inflammatory disease that targets the spinal cord and optic nerves.

Marignier et al. 2010 [44]	To study and discuss the prevalence, development, diagnosis, and management of Devic's neuromyelitis optica (DNMO).	Systematic review	This study showed that AQP4 antibodies are vital, specific biomarkers linked to the development of DNMO.

Höftberger, Rosenfeld, and Dalmau 2015 [45]	To provide an update on paraneoplastic neurologic syndromes and examine their relationship with tumors and different types of immune responses.	Meta-analysis	Paraneoplastic neurologic syndromes represent a diverse group of disorders caused by changes in the immune response. Early recognition of these conditions substantially assists in their treatment.

Honnorat et al. 2009 [46]	To examine the association between paraneoplastic neurological disorders and anti-CV2/CRMP5 and anti-Hu antibodies.	Longitudinal study	This study reported numerous cases of uveo-retinal symptoms, chorea, cerebellar ataxia, and LEMS among patients positive for anti-CV2/CRMP5 antibodies.

Irani et al. 2010 [47]	To examine the clinical spectrum of antibody-mediated CNS disorders while focusing on limbic encephalitis, Morvan syndrome and acquired NMT.	Systematic review	This study linked LGI1 and CASPR2 to neurological conditions, such as limbic encephalitis, Morvan syndrome, and acquired NMT.

**Table 3:** Antibodies/Autoantibody Targets and Associated Diseases.

Antibody/Autoantibody Targets	Association/Disease	Reference
SOX1	PND	[43]
Ma2/Ta	PND	[44]
PCA-2	PND	[43]
CV2	PND	[44]
PNMa1	PND	[48, 49]
NMDA	Encephalopathic autoimmune disorder	53
GABA	Encephalopathic autoimmune disorder	54
LGI1	Encephalopathic autoimmune disorder	[39, 50]
Anti-GAD antibodies	Stiff person syndrome	[44, 56]
Anti-TG2, TG3, and TG6 antibodies	CD, Opsoclonus-myoclonus, LEMS, MG, and NMT	[41, 42, 56]
CACNA1C and CACNB2	Psychotic disorders, MDD, ASD, ADHD, and OCD	[46, 57, 58]

## List of Abbreviations

Ab: Antibody
ADHD: Attention-deficit/hyperactivity disorder
AMAN: Acute motor axonal neuropathy
ANNA-2: Type 2 anti-neuronal nuclear antibody
ANNA-3: Type 3 anti-neuronal nuclear antibody
Anti-GM1: Anti-ganglioside member 1
Anti-Jo1: Anti-histidyl-tRNA synthetase
Anti-Hu/ANNA-1: Anti-neuronal nuclear antibody type 1
Anti-NXP2: Anti-nuclear matrix protein 2
Anti-OJ: Anti-isoleucyl-tRNA synthetase
Anti-PL7: Anti-threonyl-tRNA synthetase
Anti-PL12: Anti-alanyl-tRNA synthetase
Anti-Ri: Anti-type 2 anti-neuronal antibody
ASD: Autism spectrum disorder
AQP4: Aquaporin-4
CACNA1C: Calcium voltage-gated channel subunit alpha 1C
CACNB2: Calcium voltage-gated channel auxiliary subunit beta 2
CANOMAD: Chronic ataxic neuropathy
CAR: Cancer-associated retinopathy
CASPR2: Contactin-associated protein-like 2
C4B: Complement component 4B
CD: Celiac disease
CNS: Central nervous system
CRMP5: Collapsin response mediator protein 5
CSF: Cerebrospinal fluid
DNER: Delta/notch-like epidermal growth factor-related receptor
DNMO: Devic's neuromyelitis optica
ERG: Electroretinography
GABA: *γ*-Aminobutyric acid
GAD: Glutamic acid decarboxylase
HLA: Human leukocyte antigen
IIFT: Indirect immunofluorescence test
LEMS: Lambert-Eaton myasthenic syndrome
LGI1: Leucine-rich glioma-inactivated 1
Ma2/Ta: Protein in the nucleoli of neuron nuclei
MAG: Myelin-associated glycoprotein
MAR: Melanoma-associated retinopathy
MDD: Major depressive disorder
MG: Myasthenia gravis
mGluR1: Metabotropic glutamate receptor 1
NMDA: N-Methyl-D-aspartate
NMDAR: N-Methyl-D-aspartate receptor
NMT: Neuromyotonia
OCD: Obsessive-compulsive disorder
PCA-1: Purkinje cell cytoplasmic antibody type 1
PCA-2: Purkinje cell cytoplasmic antibody type 2
PNDs: Paraneoplastic neurological disorders
PNMA1: Paraneoplastic antigen MA1
PNMA2: Paraneoplastic antigen MA2
SOX1: SRY-Box 1
SAN: Sensory ataxic neuropathy
TG2: Tissue transglutaminase type 2
TG3: Tissue transglutaminase type 3
TG6: Tissue transglutaminase type 6
VGCCA: Voltage-gated potassium channel complex
Zic4: Zic family member 4.
